# Discovery of alkaline laccases from basidiomycete fungi through machine learning-based approach

**DOI:** 10.1186/s13068-024-02566-6

**Published:** 2024-09-11

**Authors:** Xing Wan, Sazzad Shahrear, Shea Wen Chew, Francisco Vilaplana, Miia R. Mäkelä

**Affiliations:** 1 Department of Microbiology, Faculty of Agriculture and Forestry, University of Helsinki, Biocenter 1, Viikinkaari 9, 00790 Helsinki, Finland; 2 Division of Glycoscience, Department of Chemistry, School of Engineering Science in Chemistry, Biotechnology and Health, KTH Royal Institute of Technology, AlbaNova University Center, Roslagstullbacken 21, 11421 Stockholm, Sweden; 3 Present Address: Department of Bioproducts and Biosystems, Aalto University, Kemistintie 1, 02150 Espoo, Finland

**Keywords:** Machine learning, Alkaline laccase, pH optimum, Prediction, Basidiomycete fungi

## Abstract

**Background:**

Laccases can oxidize a broad spectrum of substrates, offering promising applications in various sectors, such as bioremediation, biomass fractionation in future biorefineries, and synthesis of biochemicals and biopolymers. However, laccase discovery and optimization with a desirable pH optimum remains a challenge due to the labor-intensive and time-consuming nature of the traditional laboratory methods.

**Results:**

This study presents a machine learning (ML)-integrated approach for predicting pH optima of basidiomycete fungal laccases, utilizing a small, curated dataset against a vast metagenomic data. Comparative computational analyses unveiled the structural and pH-dependent solubility differences between acidic and neutral-alkaline laccases, helping us understand the molecular bases of enzyme pH optimum. The pH profiling of the two ML-predicted alkaline laccase candidates from the basidiomycete fungus *Lepista nuda* further validated our computational approach, showing the accuracy of this comprehensive method.

**Conclusions:**

This study uncovers the efficacy of ML in the prediction of enzyme pH optimum from minimal datasets, marking a significant step towards harnessing computational tools for systematic screening of enzymes for biotechnology applications.

**Graphical Abstract:**

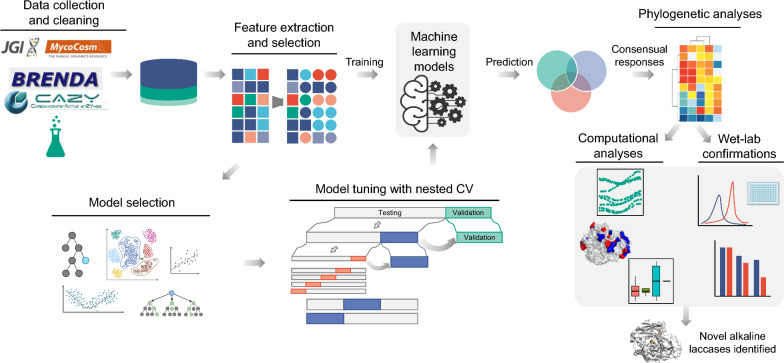

**Supplementary Information:**

The online version contains supplementary material available at 10.1186/s13068-024-02566-6.

## Background

Together with the rapid technological advancements, such as whole genome sequencing across different species, the exponentially increasing number of metadata is becoming available on hourly basis. Hence, scientists are more eager than ever to build informative and predictive models for better understanding the biological processes in a more efficient manner [1].

Enzymes, as catalysts of biological processes, are not only central to understand life at the molecular level, but also tools for different industrial, medical, and environmental applications. The engineering of enzymes to enhance their properties, such as stability, specificity, and activity, requires a deep understanding of the relationship between enzyme structure and its function [2]. While the vast diversity of enzymes and the intricate nature of their catalytic mechanisms address a huge challenge in molecular engineering, the growing need of efficient biocatalysts has increasingly encouraged researchers to harness the power of machine learning (ML) to accelerate the discovery and optimization of enzymes for diverse applications [3–5].

Laccases (EC 1.10.3.2, *p*-diphenol:oxygen oxidoreductase) belong to the family of multi-copper oxidases, and they are widely distributed across species including bacteria, archaea, fungi, lichens, and even insects [6]. In the Carbohydrate-Active enZyme (CAZy) database (www.cazy.org, [7]), laccases sensu stricto are categorized to Auxiliary Activity (AA) family 1, sub-family 1 (AA1_1). These enzymes have four copper atoms in two spatial centers, where T1 copper is considered as the substrate oxidation site, and one type-2 and two type-3 coppers form a cluster (T2/T3) to react with and reduce molecular oxygen to water [8]. Because of this, laccases are often regarded as green catalysts, and they are not only able to oxidize various phenolic compounds, but their substrate range can also be extended to nonphenolic compounds with the help of small mediator molecules that act as electron carriers [9]. Therefore, laccases are particularly valuable for their potential in a wide range of biotechnological applications, such as bioremediation, biomass processing, and synthesis of biopolymers [6].

The pH optima and working pH ranges of laccases, varying from highly acidic to extreme alkaline [10–12], determine their applicability in different industrial processes. In the past years, researchers have succeeded to either engineer well-characterized laccases through extensive directed evolution approaches [9, 11] or build up screening methods to identify new laccases by single genome [10] and detailed phylogenetic analyses [13] to meet the specific industrial requirements regarding the working pH ranges of laccases. However, these laccase discovery approaches centered with wet-lab experiments are often labor-intensive and time-consuming, and individual genome-wide analyses hardly match the pace of the genomic information explosion. Therefore, a great variety of laccase candidates present in nature remain untapped for biotechnology applications.

Recent advances in computational methods, particularly ML, offer a promising alternative for the rapid prediction and analysis of enzyme properties. Supervised, semi-supervised and unsupervised models have been developed very recently to predict enzyme properties, such as kinetic parameters [14] and substrate specificity [15–17]. Yet, the lack of large amount of high-quality training data has hindered the prediction accuracy in many ML tasks, due to the innate difficulties in benchmark data acquisition from the laboratories. Adaptive and accurate computational approaches are needed to address this gap and to enable large-scale enzyme property prediction in the future.

In this study, we present a comprehensive approach centered with ML and its applicability in the prediction of pH optimum using basidiomycete laccases as an example, leveraging a small but high-quality training data from multiple sources to predict massive metagenomic data. By compiling a comprehensive clean dataset and applying several ML algorithms, we discovered patterns and features that contribute to the prediction of the pH optimum of laccases, a property of high interest for industrial applications. Our approach stressed the significance of a proper model selection to mitigate the limitations of each model on small datasets. Moreover, we highlighted the relevance of amino acid composition and phylogenetic information for predicting the pH optimum of laccases. Through computational analyses, we compared pH-dependent solubility profile and structural differences between acidic and neutral-alkaline laccases, providing insights into the biological basis that determine enzyme pH optimum. Most importantly, we validated our ML prediction on the alkaline laccases by conducting in vitro pH profiling on two candidates originated from the saprotrophic fungus *Lepista nuda*.

## Materials and methods

### Data collection and preprocessing

To construct a high-quality benchmark dataset for model training, we performed extensive literature searches and collected 39 characterized laccases which pH optimum were experimentally determined, including mutated laccases and laccases from non-fungal species as positive training instances (Table S1) together with 16 in-house characterized laccase candidates from various basidiomycete fungi (unpublished).

For the testing dataset, we extracted the amino acid sequences which have been annotated as AA1_1 family laccases from all publicly available basidiomycete fungal genome sequences from JGI MycoCosm database (https://mycocosm.jgi.doe.gov, data retrieved: 31 August 2023). The duplicate sequences were removed using seqkit rmdup [18]. Secretion signal peptides were predicted with SignalP 6.0 [19] and removed from the precursor sequences.

### Feature extraction and selection

We extracted five different categories of features: phylogenetic information, biochemical features, primary and secondary protein structure-based features, as well as features associated with substrate binding. In total, 42 initial features were obtained (Table S1). Through feature transition, characteristic features are one-hot encoded and missing values from numeric features are replaced with − 1. Thus, after all, 89 numeric features were obtained and used in the ML regression (data not shown).

A phylogenetic tree was built based on all 2019 clean laccase amino acid sequences. A FASTA file containing these sequences was used to create multiple sequence alignment (MSA) by MUSCLE Super5 algorithm [20]. Laccases from non-basidiomycete fungi functioned as the out-group. To remove spurious sequences and poorly aligned regions from the resultant MSA, we used trimAl with default settings. An approximately maximum-likelihood phylogenetic tree (data not shown) was generated from the trimmed MSA with FastTree (data not shown, [21]). The branch lengths of each leaf nodes were extracted from the tree file. We also performed an intensive literature search to assign the natural habitats for the laccase host species that appear in the training dataset.

Fungal laccases are glycosylated proteins, and glycosylation plays a crucial role in the enzyme activity [22]. Therefore, we included the presence of putative N-glycosylation sites in the laccase amino acid sequences predicted by NetNglyc 1.0 [23] as a feature for the datasets. A positive result was considered only when the jury agreement is 9 out of 9 and indicated as 1 in the feature column, while a negative result is indicated as 0 (Table S1).

As laccase activity and pH profile are largely substrate dependent, for the 16 in-house characterized laccases, we also studied their potential for oxidization of 2,6-dimethoxyphenol (2,6-DMP) in silico using molecular docking with SwissDock [24]. The numeric rank of the predicted energy used for 2,6-DMP binding to the putative T2/T3 substrate binding pocket of laccases was used as such. If the predicted docking position did not align with the putative substrate binding areas, value 0 was used.

Theoretical molecular weight and isoelectric point of each laccase were calculated using the ProtParam module from Biopython’s SeqUtils package, available in a python script aa2csv.py (https://raw.githubusercontent.com/xing1wan/scripts/main/aa2csv.py).

Mature laccase sequences were numerically represented using per residue amino acid composition and per group composition. The amino acid composition for each laccase was calculated to determine the relative abundance of the canonical amino acids. The amino acids were also computed in groups according to their side chains, namely positively (basic) or negatively charged (acidic), polar or non-polar, and aliphatic or aromatic, to allow the models learn the importance of amino acids beyond the individual amino acid composition. The sequences were transformed to numeric representation using the ProtParam module from Biopython’s SeqUtils package available in the python script aa2csv.py, yielding a 25-dimensional feature vector.

Important secondary structures including coil, helix and strand of each laccase were calculated using psiPRED [25]. Percentages of coil, helix and strand are extracted from the generated ss2 files using a python script ss2csv.py (https://raw.githubusercontent.com/xing1wan/scripts/main/ss2csv.py).

The monomer model of AlphaFold2 (AF2, [26]) was used to generate secondary structures from the mature amino acid sequences from the training dataset. Based on our previous unpublished findings, we speculated the amino acids in close proximity to both T1 and T2/T3 copper centers are responsible for substrate binding of basidiomycete fungal laccases. *Panus rudis* (JGI Protein ID: 1594824) was used as a model basidiomycete laccase protein to identify the substrate binding amino acids near the T1 and T2/T3 copper centers. Its putative substrate binding areas was calculated using a SwissDock-based method, as previously described [27]. The positions of the substrate binding residues of *P. rudis* laccase were estimated to be within 5 Å from the 2,6-DMP, which was predicted to form hydrogen bonds with the protein with low energy (Fig. S1). The obtained MSA, as described above, was then used to localize these reference residues of the *P. rudis* laccase, and as the input file to identify the amino acids from putative substrate binding areas for the rest of the laccases using a python script select_sbs.py (https://raw.githubusercontent.com/xing1wan/scripts/main/select_sbs.py). The occurrences of substrate binding amino acids were calculated into groups, i.e., positively (basic) or negatively charged (acidic), polar or non-polar, and aliphatic or aromatic amino acids.

### Learner selection

Because the model performance can largely depend on the dataset, we screened all available regression learners from the original mlr3 package, as well as mlr3learners and mlr3extralearners packages, to select suitable models for the existing training dataset. The performances from untuned models were calculated using regr.rmse function from the R mlr3measures package.

The performances were calculated by root mean squared error (RMSE), as defined in Eq. (1):

1 $${\text{RMSE}} = \sqrt {\frac{1}{n}\mathop \sum \limits_{i = 1}^{n} \left( {y_{i} - \hat{y}_{i} } \right)^{2} } ,$$

where *n* is the number of observations in the validation set, *y*_*i*_ is the actual value of the observation, and $\hat{y}_{i}$ is the predicted value from the model.

### Training routine and optimization

The ML methods were implemented with mlr3 and derived packages [28]. Learner training was carried out on R (version 4.3.2). Search spaces were defined based on the default parameters specified by mlr3tuningspaces for each chosen learner. To identify the best hyperparameters and evaluate the performance of the derived model, a nested cross-validation (CV) procedure was implemented using the mlr3tuning package. The inner CV focused on hyperparameter tuning, while the outer CV was employed to evaluate the learner’s performance using the optimal hyperparameters identified in the inner CV. The final model was built by using the optimal hyperparameters applied on the entire training data, which includes both training and validation datasets. Details regarding the search space of the grid search and the optimized hyperparameters based on nested CV results are listed in Table S2.

### Evaluation of model performance

The performance of the models was evaluated through two standard methods: nested CV and an independent test. The nested CV procedure offers an unbiased estimation of a model performance, presenting a more reliable assessment compared to classical CV [29]. Considering the relatively limited size of our training set, we performed nested CV with five inner loops and three outer loops. The independent test used a separate dataset, which was completely distinct from the training data.

The RMSE values obtained from each fold of the CV were then averaged to produce a single performance metric representing the model’s overall predictive accuracy. The average RMSE shows how well the model is likely to perform on unseen data with its tuned hyperparameters. A lower RMSE value indicates a model that predicts more closely to the actual values.

The model behaviors were assessed by learning curves with an incremental learning sample size. The learning sample sizes were increased from 20 to 100% by 2% of the training dataset and the tuned model performance was evaluated with RMSE on the training data subsets and independent validating dataset. Decreasing RMSE values and a converging trend of the two learning curves indicate a near optimality.

Permutation feature importance was calculated using the FeatureImp function with 100 repetitions from the R iml package on the optimal models trained with the full training data. Median importance, 5% quantile of importance values from the repetitions and 95% quantile were calculated. SHapley Additive exPlanations (SHAP) method was used to calculate the SHAP feature importance through the Shapley function from ilm package with sample size of 50 on the optimal models trained with the full training data. The average of absolute SHAP values was calculated to reveal the global importance of each feature on the model performance. The weights of each feature types of the top 20 most important features and SHAP features was also compared.

### Selection of candidate laccases for in vitro validation

Laccases from testing dataset with target predicted pH optimum of pH ≥ 7.0 were analyzed further. The intersect of the prediction results from at least three learners were considered viable. Moreover, the phylogenetic relationship between the candidates and known laccases with target pH optimum was calculated by the cophenetic distances and only the cophenetic distances between 1.1 and 1.5 were considered. The distribution of individual prediction responses was analyzed to find out a unified prediction verdict.

### Functional characterization

Candidate laccase-encoding genes *lccA* and *lccB* from the basidiomycete fungus *L. nuda* were codon-optimized for *Pichia pastoris* and synthesized and cloned into pPICZαA expression vectors at EcoRI and NotI sites (GenScript Biotech, Leiden, The Netherlands). The recombinant plasmids were amplified in *E. coli* DH5α and linearized at PmeI site prior to genomic integration in *P. pastoris* X-33 via electroporation. Recombinant laccase production in *P. pastoris* was carried out in shake flasks according to a previous study [12]. The cell-free culture supernatants were concentrated using 10 kDa molecular weight cutoff (MWCO) membrane in Amicon® nitrogen gas-pressured stirred cell (Merck, Rahway, New Jersey, USA), followed by concentration with Amicon® Ultra centrifugal filter units with a 10-kDa MWCO membrane (Merck, Rahway, New Jersey, USA). The approximately 400-fold concentrated laccase crude extracts were stored at 4 °C.

The pH optima of the recombinant *L. nuda* laccases were determined by measuring the oxidation of 2,6-DMP (Sigma-Aldrich, St. Louis, USA) across various pH values at 476 nm [30] in a Spark microplate reader (Tecan, Männedorf, Switzerland) in either McIlvaine or Britton–Robinson buffer adjusted for pH in a range from pH 2.0 to pH 8.0 or from pH 8.0 to pH 12.0, respectively, with increments of 0.5 pH units. The oxidation of 2,2′-azino-bis(3-ethylbenzothiazoline-6-sulfonic acid) (ABTS) was measured at 420 nm [31] in McIlvaine buffer from pH 2.0 to pH 7.0 with increments of 1 pH units. The total protein concentration of the protein concentrates was determined by bicinchoninic acid method using Pierce™ BCA protein assay kit (Thermo Scientific, Waltham, MA, USA) according to the manufacturer’s instructions. The laccase activity is presented as nkat/mg of total protein. Additionally, the residual laccase activity of the recombinantly produced *L. nuda* laccases was determined after incubation in the Britton–Robinson buffer at the pH optimum of *Ln*LccA (JGI Protein ID: 1172164, pH 10.0) and *Ln*LccB (JGI Protein ID: 1268943, pH 9.0) for 1 to 24 h at 22 °C. All measurements were performed in triplicate.

### Computational characterization

The AF2-predicted structures with the highest average pLDDT score were used for all following in silico analyses. The locations of the copper ions from the two copper centers of laccases in the predicted laccase structures were identified by superimposing the predicted structures with the X-ray crystal structure of the basidiomycete *Trametes trogii* laccase 2HRG [32] from the Protein Database (PDB, www.rscb.org).

Protein structural pairwise comparison was performed using candidate laccases selected in this study against previously characterized laccase *Or*Lac1 from the basidiomycete fungus *Obba rivulosa*, which has optimal pH at pH 3.5 [12], using script list_unaligned_resi.py (https://raw.githubusercontent.com/xing1wan/list_unaligned_resi/main/list_unaligned_resi.py). Protein surface residues were selected using script findSurfaceResidues.py (https://raw.githubusercontent.com/Pymol-Scripts/Pymol-script-repo/master/findSurfaceResidues.py) with a cutoff of 30 Å for high specificity in identifying only the most exposed residues. The surface amino acids were also computed in positively or negatively charged, polar or non-polar, and aliphatic or aromatic groups using the python script aa2csv.py.

Furthermore, the laccase candidates from the ML pipeline were analyzed by CamsolpH [33] in comparison with previously characterized laccases from the full training dataset to visualize the correlation between their pH-dependent solubility and potential pH optimum. CamsolpH calculates the CamSol intrinsic solubility profile, and the protein solubility of its unfolded state at varying pH values. The CamSolpH resultant solubility profile contains one score per residue in the given protein sequence. Protein regions with scores larger than 1 indicate highly soluble regions, while scores smaller than − 1 poorly soluble ones.

## Results

### Performance evaluation and model selections

To tune the selected models, we compiled a dataset of 55 basidiomycete laccases, which pH optimum has been determined experimentally, from BRENDA and CAZy databases, and from our in-house database (unpublished). To prevent data leakage, these 55 characterized laccases were split randomly into training data (*n* = 50) and validation data (*n* = 5), with the guaranteed presence of both acidic and alkaline laccases in both datasets (Table S1). Consequently, we prepared an additional dataset of 1964 protein sequences which were annotated as members of CAZy AA1_1 laccase subfamily from the published basidiomycete genomes available on the JGI MycoCosm. This testing dataset was used to examine the potential advantages of applying ML models in selection of alkaline laccases.

To select suitable models to tune, we tested and compared 32 native learners on both the training and validation datasets, and their performances varied drastically on both datasets as the RMSE values from the training dataset (RMSE_tra_) ranged from 0.10 to 4.02 and from the validation dataset (RMSE_val_) ranged from 0.68 to 85.42 (Table S3). Low RMSE_tra_ but high RMSE_val_ indicate the model being overfitting. Therefore, we selected 1.30 as the cutoff RMSE_val_ value according to the performance of native learner. The maximum difference between the performances of the training and validation datasets was set to 0.30 to avoid over- or under-fitting.

**Fig. 1:**
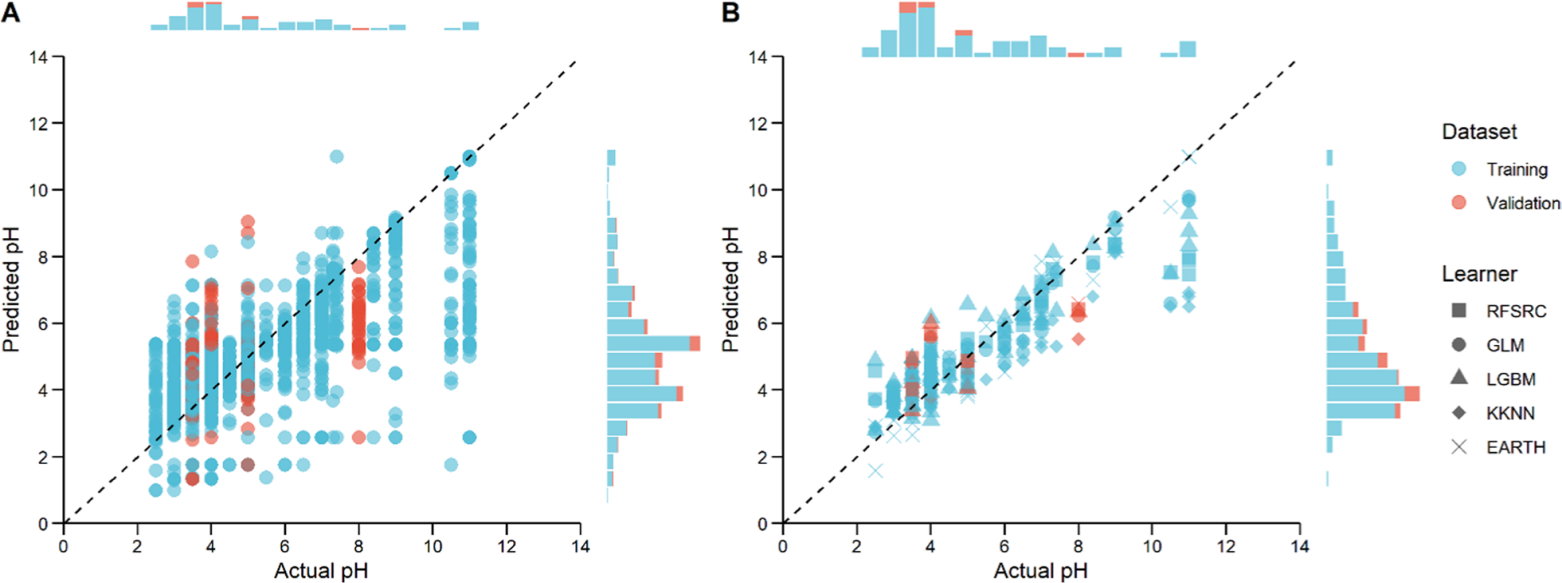
Learner performances. Scatter plots comparing the pH optimum values predicted by the native regression learners and the experimentally determined pH optimum of the 55 candidate laccases. Results generated by all 32 native learners available in the mlr3 and relative packages (**A**) and by the five native learners suitable for our datasets (**B**). Distributions of the log transformed data are show in the bar charts on top or to the right of the plots. A small constant value (1e−6) was added to all occurrences to prevent zeroizing values. Extreme outliers with values outside of the pH range 0–14 are eliminated from the plot. Distribution and pH values from training dataset and validation dataset are indicated in blue and red, respectively. Dashed line represents a linear regression line where the predicted pH optima and experimentally determined pH optima are uniform

Selection of reliant native models to start with can significantly increase the efficiency of model tuning, since the performance of models is inevitably data dependent. The variability in performance among the native learners tested in this study reveals the intricate nature of predicting enzyme activities in broad range of pH values (Fig. 1A). Given the small nature of the training dataset in this study, only random forest for survival, regression, and classification learner (RFSRC), boosted generalized linear regression model (GLM), light gradient-boosting machine regression learner (LGBM), weighted k-nearest-neighbor regression model (KKNN), and enhanced adaptive regression through hinges learner (EARTH) were selected for further tuning (Fig. 1B). All selected native learners tend to under-predict the optimal pH values for extremely alkaline laccases with experimentally validated pH optimum above pH 10.0.

### Model interpretation

**Fig. 2:**
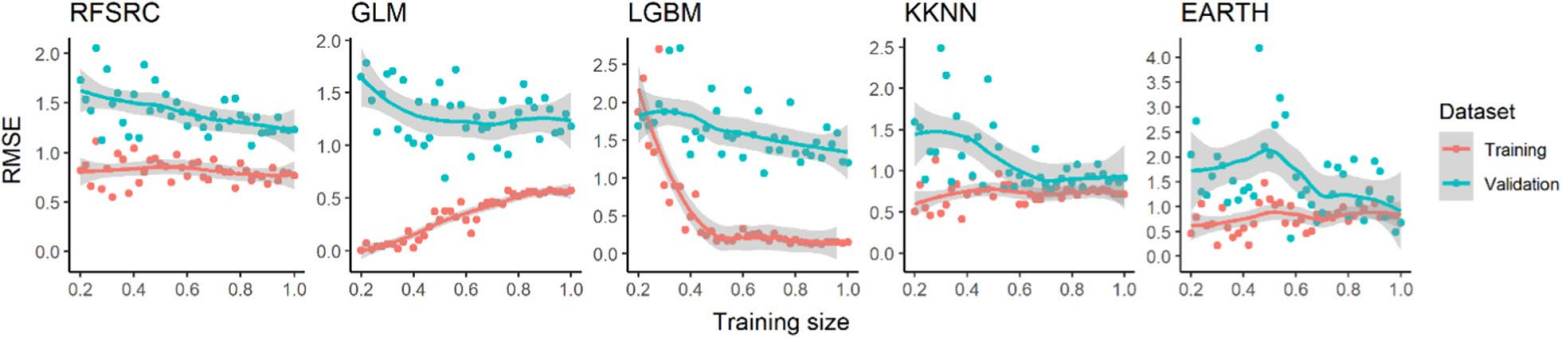
Learning curves for tuned models RFSRC, GLM, LGBM, and KKNN, and the native EARTH. Training dataset and validation dataset are shown in red and cyan, respectively. The grey areas show the local polynomial regression fitting computed with loess function in the R stats package

In this study, we reached near optimal for two of the selected models, and the other models performed significantly better after tuning than at their native settings. Most of the optimal models tend to overfit to some extent as shown in Fig. 2, where RMSE_val_ are overall higher than the corresponding RMSE_tra_ from the same learner.

The RMSE_tra_ increases while the RMSE_val_ decreases with an overall higher RMSE_val_ suggesting that the model is overfitting less as it is trained on larger subsets of data, as seen from GLM. Initially, the model might be capturing noise or overemphasizing patterns specific to the smaller training sets, leading to lower RMSE_tra_. As the training data increase, the model likely reduces overfitting, which aligns its performance more closely between training and validation datasets. While the LGBM model appears to learn effectively over a given training dataset, its performance stabilizes when given larger training data. The ongoing decrease in RMSE_val_ suggests that further optimization is possible, either by increasing the model complexity or broadening the hyperparameters for tuning. The tuned RFSRC model has a good initial fit on training data, as the RMSE_tra_ is low and stabilizes, this could mean that the model is sufficiently complex to capture the underlying patterns in the training data without requiring much additional data to improve its fit. The convergence of RMSE_tra_ and RMSE_val_ with lower RMSE_tra_, suggests that the model complexity might be near optimal for the given task. Nevertheless, similar to the LGBM model, the continuing decreasing RMSE_val_ suggests that slight justification of the model RFSRC might still be needed. The tuned KKNN and untuned EARTH models showed near optimal complexity with minimal overfitting, as it has general stabilized RMSE_tra_, decreasing RMSE_val_ and converging trend.

**Fig. 3:**
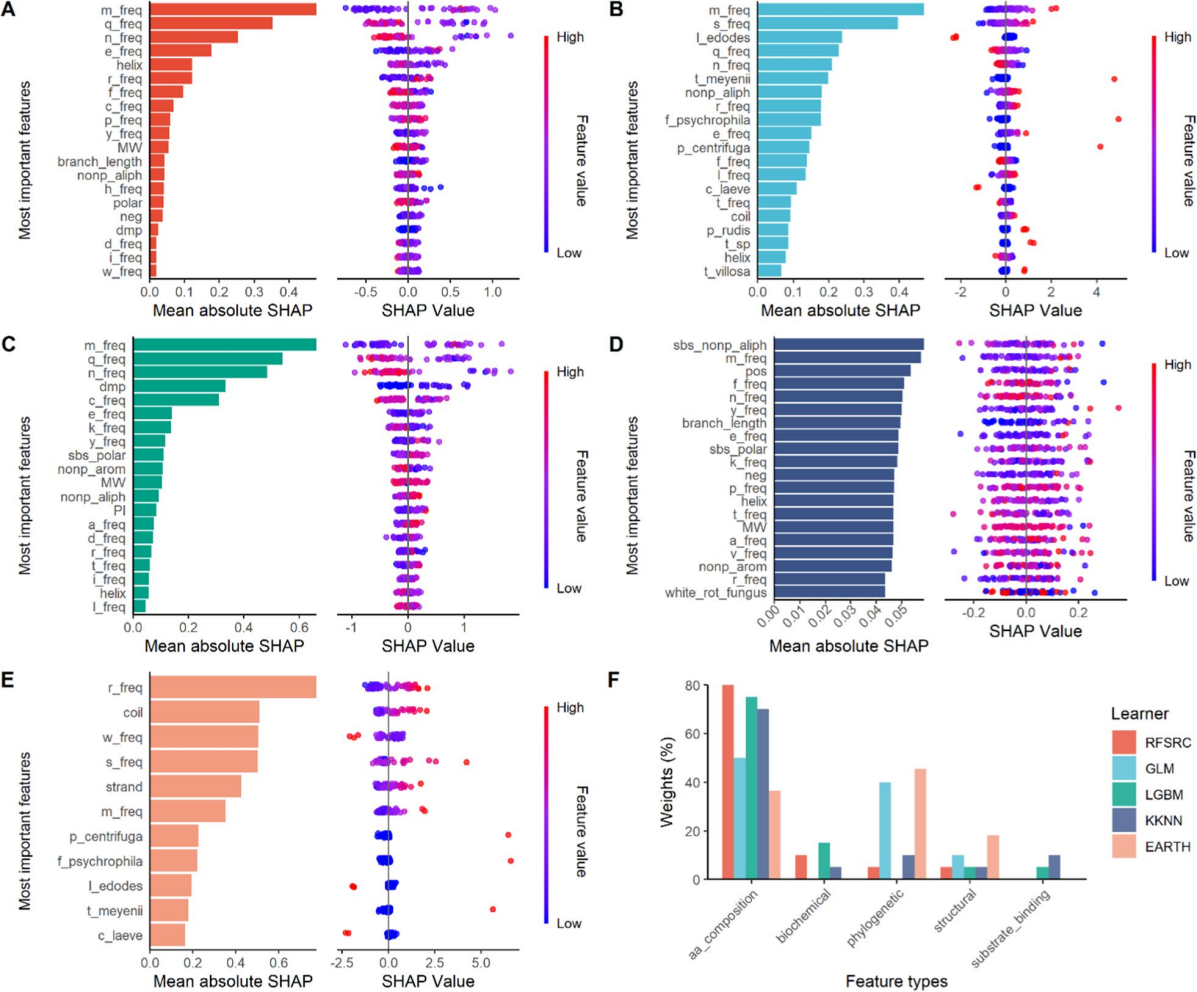
SHAP feature importance. The most important SHAP features, and their corresponding SHAP summary plots are obtained from the four tuned models **A** RFSRC, **B** GLM, **C** LGBM, **D** KKNN, and one native model **E** EARTH. In the summary plots, each point is a Shapley value for a feature and an instance of the corresponding feature, with red indicating high values and blue indicating low values. **F** The weights of each feature types on the prediction decisions. Feature types are found in Table S1

To better understand how data features affect the model prediction, we employed permutation and SHAP feature importance techniques. All five selected learners performed differently and replied on different types of features to make the prediction. From both permutation and SHAP methods, the most important features for predicting pH optimum by the tuned learners belong to amino acid composition, as they showed higher importance scores than other feature types (Fig. 3, S2). Nevertheless, for models GLM, EARTH, and KKNN, around 40–60% of the host-related features also contributed to the models’ final verdicts (Fig. S2, Fig. 3F). Biochemical properties, such as putative binding efficiency of reference substrate 2,6-DMP and post-translational modification, and information on protein secondary structure may be minimal but cannot be completely neglected either.

### Prediction of laccases with an alkaline pH optimum

The models were set to their optimal hyperparameters prior to the prediction. The native learner EARTH pre-tuning already outperformed the other selected learners, and any changes in the parameters resulted in great loss in the model accuracy. Therefore, this learner was used with default hyperparameters in the prediction of the pH optimum on test dataset.

The tuned RFSRC model predicted 39 laccases that potentially have pH optima higher than pH 7.0, from basidiomycete genomes. The tuned GLM model predicted 103, tuned LGBM 171, tuned KKNN 139 and native EARTH model 305 laccases that possibly work at neutral-alkaline pH (Table S4). To reduce the model bias, we considered the 75 common candidates that were predicted by at least three learners.

**Fig. 4:**
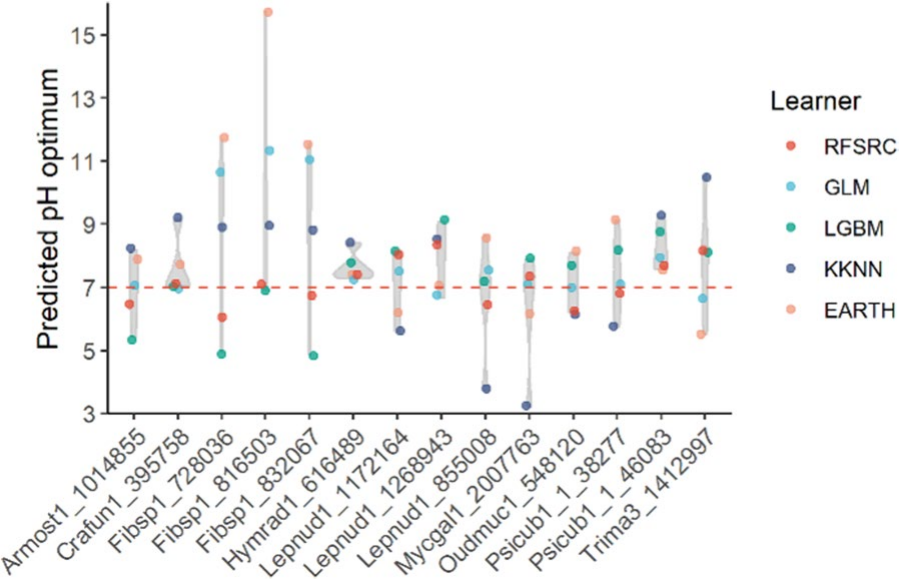
Distribution of predicted pH optimum from the five optimal models of the 14 candidate laccases. Red dashed line at pH 7.0 indicates the division of putative acidic and alkaline laccases. Information of laccase presented as fungal species abbreviations and protein IDs in the *x*-axis can be found in Table S1

For the experimental feasibility and proof of concept, a smaller subset of 14 laccase candidates (Fig. S3) was analyzed further, as they had moderate cophenetic distances to characterized alkaline laccases from basidiomycete fungi with pH optimum above pH 10.0 (1.1 < CD < 1.5) but longer distances to known basidiomycete laccases with pH optimum at pH 7.0 (Fig. S3**,** Table S1). We also analyzed the individual prediction responses of the resultant candidates to select the most likely candidates with consensual prediction responses from all five optimal models. After thorough examinations, we selected *L. nuda* laccases *Ln*LccA and *Ln*LccB for biochemical characterization, although the candidates from *Armillaria solidipes* (JGI Protein ID: 1014855), *Crassisporium funariophilum* (JGI Protein ID: 395758), *Hymenopellis radicata* (JGI Protein ID: 616489), *Oudemansiella mucida* (JGI Protein ID: 548120), and *Psilocybe cubensis* (JGI Protein IDs: 38277 and 46083) may potentially also have alkaline pH optimum (Fig. 4). The complete prediction responses for the 75 candidate laccases can be found in Table S5.

### Computational characterization

**Fig. 5:**
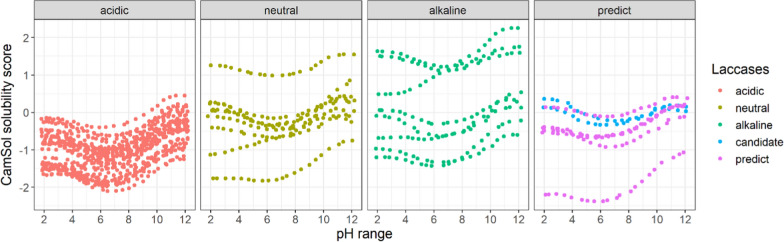
CamsolpH solubility profiles. Laccases from training dataset are shown according to their pH optimum. The candidate *Ln*Lccs are shown in blue, and the other six basidiomycete fungal laccases predicted to also have optimal pH above 7.0 are shown in purple

Although the origins of laccases used in the training dataset of this study are from various microbes, their CamSolpH profiles could still demonstrate pH-dependent correlation among the laccases with different optimal pH. The CamSolpH solubility profile reveals a positive correlation of the two *L. nuda* laccases with other characterized neutral-alkaline laccases, as they show higher solubility over a wide pH range than the acidic laccases from various species (Fig. 5). The solubility profiles of other candidate laccases also follow the same pattern, except for *P. cubensis* (JGI Protein ID: 38277, Fig. 5, Table S6).

**Fig. 6:**
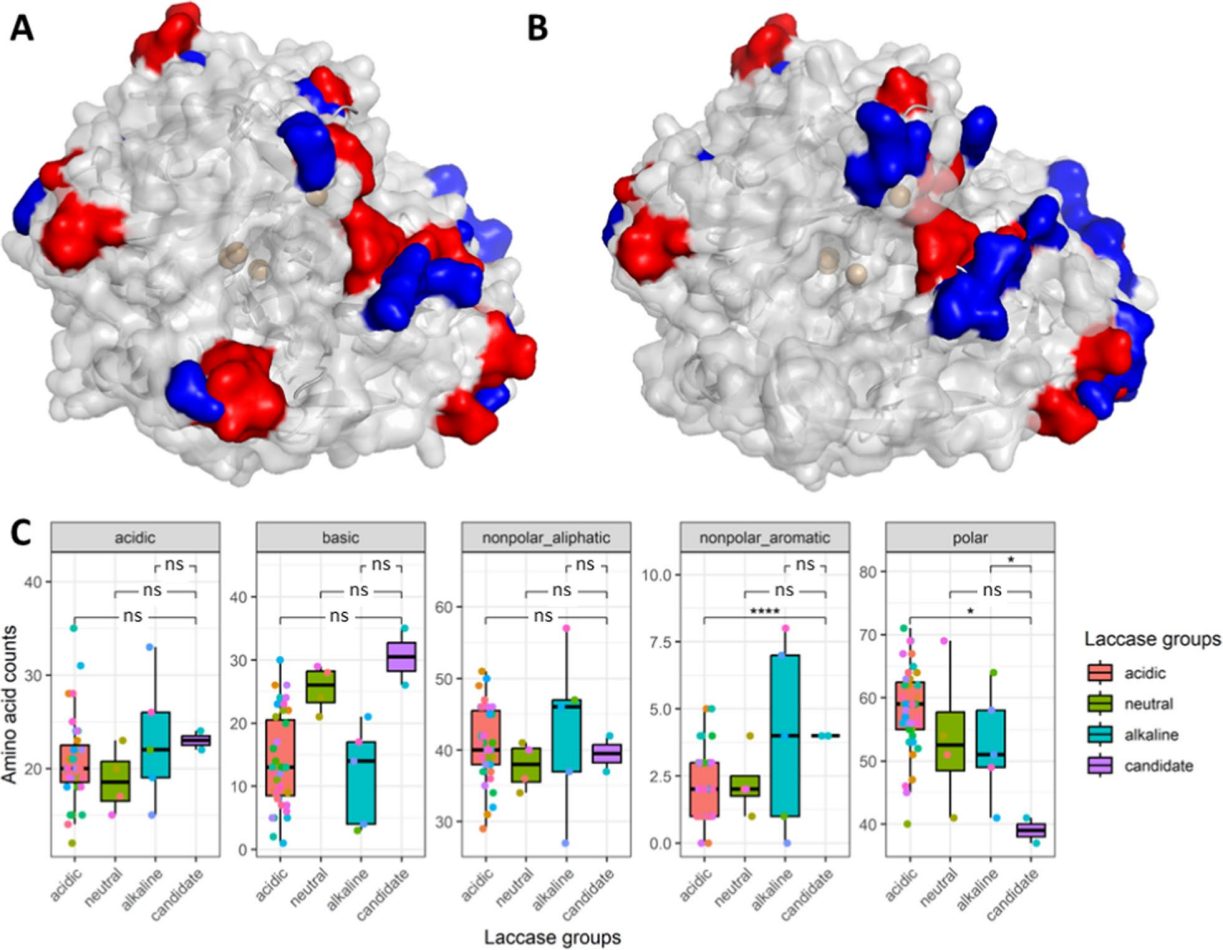
Computational analyses of laccase surface amino acids. Comparison between the acidic laccase *Or*Lac1 from *O. rivulosa* [12] and alkaline laccase candidates *Ln*LccA (**A**) and *Ln*LccB (**B**) from *Lepista nuda*. Reference laccase 2HRG from *Trametes trogii* shown in grey cartoon to depict the copper centers (brown spheres). *Ln*LccA, *Ln*LccB and *Or*Lac1 are shown in surfaces, with aligned residues shown in grey, and unaligned residues from *Ln*Lccs and *Or*Lac1 in blue and red, respectively. **C** Boxplots presenting the amino acid counts of the surface residues of all characterized laccases from the training dataset with various predicted pH optimum and candidate laccases *Ln*Lccs (Table S8). Statistical significances were calculated between *Ln*LccA and *Ln*LccB and other characterized laccase groups using pairwise *t*-test. *0.0001 < *p*-value < 0.05; ****p-value < 0.0001; ns, not significantly different with *p*-value > 0.05

The AF2-predicted structures of mature *L. nuda* laccases were superimposed with a reference laccase 2HRG from the white-rot fungus *T. trogii* [32] to reveal their theoretical copper centers. To identify the possible amino acid regions determining the pH optimum of the enzymes, the alpha carbons of the structures of *L. nuda* laccases were aligned with those of *O. rivulosa* Lac1 (*Or*Lac1), whose pH optimum has been experimentally determined to be at pH 3.5 [12] at an atomic distance threshold of 2 Å. The surface amino acids seem to have major impact on the predicted laccase pH optimum, as their spatial positions vary the most between the acidic and alkaline laccases (Fig. 6A, B). All aligned residues between *Or*Lac1 and *Ln*Lccs are listed in Table S7, with Euclidean distances between their corresponding alpha carbons.

Based on this observation, we next examined whether the surface residues of *Ln*Lccs showed any correlation with those of other basidiomycete laccases. The composition of total surface amino acids revealed that *Ln*LccA and *Ln*LccB possess more non-polar aromatic amino acid residues and much fewer polar residues than those basidiomycete laccases with acidic pH optima with significant differences (Fig. 6C). The two *Ln*Lccs possess significantly lower number of polar residues on their protein surface compared to other laccases with different pH optimum, potentially indicating a unique enzyme-specific property. Nevertheless, in terms of non-polar aromatic residues on the surface, the positive correlation of the two *Ln*Lccs to alkaline laccases but negative correlation to acidic and neutral laccases might give a hint on how surface amino acids contribute to the enzyme pH optimum.

### Biochemical characterization

**Fig. 7:**
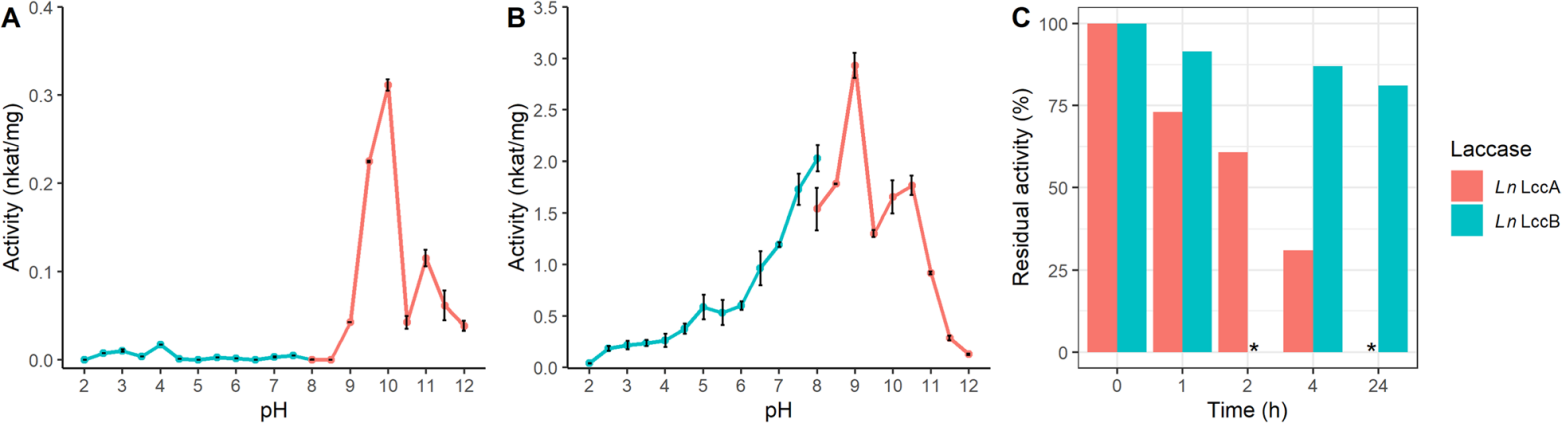
Effect of pH on the activity of recombinant *Lepista nuda* laccases **A** *Ln*LccA and **B** *Ln*LccB towards 2,6-DMP. Activities measured in McIlvaine and Britton–Robinson buffers are depicted by cyan and red lines, respectively. Note that the specific activities of *Ln*LccA and *Ln*LccB are presented in different scales. The error bars represent standard deviation from the triplicate measurements. **C** The residual activity of the two *L. nuda* laccases after incubation at their pH optimum for 0–24 h at 22 °C. Standard deviations of the triplicate measurements were below 0.5%. *LnLccA activity was not measured at 24 h, and *Ln*LccB activity was not measured at 2 h

To experimentally validate the performance accuracy of our ML pipeline, we determined the pH dependence of the activity of *Ln*LccA and *Ln*LccB for the oxidization of phenolic substrate 2,6-DMP and nonphenolic substrate ABTS (Fig. 7, S4). *Ln*LccA showed a working pH spectrum at alkaline pH values, from 9.0 to 12.0, with the highest activity 0.31 nkat/mg at pH 10.0 (Fig. 7A). *Ln*LccB was highly active throughout a much wider pH range from pH 2.5 to pH 11.5, and its optimum pH was determined at pH 9.0 (2.93 nkat/mg, Fig. 7B), showing that both *Ln*LccA and *Ln*LccB are alkaline laccases. Moreover, *Ln*LccB was very stable at its pH optimum, as it retained more than 80% activity after incubation at pH 9.0 for 24 h, while *Ln*LccA showed only 30% activity after 4 h incubation at pH 10.0 (Fig. 7C). When using nonphenolic compound ABTS as substrate, both *L. nuda* laccases exhibited also acidic pH optimum at pH 4.0 (Fig. S4).

## Discussion

Alkaline laccases are ideal biocatalysts, e.g., for delignification of alkaline-pretreated lignocellulosic feedstocks or degradation of recalcitrant dye compounds [34]. However, most of the characterized basidiomycete laccases have pH optimum in acidic pH range, which restricts their use in alkaline conditions in industrial applications. The traditional experimental methods for the discovery of novel alkaline laccases, including site-directed mutagenesis and directed evolution [35, 36], are often labor-intensive and time-consuming. At the same time, the tremendous potential to exploit the biodiversity of native laccases from the basidiomycete fungal genomes has largely been neglected. Fortunately, the advancements in computational methods such as ML illuminate a promising path towards efficient and reliable prediction and analyses of enzyme properties.

In this study, we generated a detailed pipeline for the targeted selection of neutral to alkaline basidiomycete laccases by integrating a robust ML algorithm to predict enzyme pH optimum. Unlike conventional laccase engineering, which relies on intensive evolutionary mutagenesis, our ML-driven strategy hugely improves the efficiency of metagenome mining on the targeted discovery of native fungal laccases with the ability to catalyze reactions at specific pH values. Our comprehensive assessments demonstrated that regression ML models, have the capacity and they outperform numerous traditional computational methods in predicting the pH optimum of laccases, further reinforcing the potential of using supervised or semi-supervised strategies in facilitation of precise enzyme selection and engineering [37].

The construction of a high-quality benchmark dataset for training and validating the model is a prerequisite to perform a successful ML prediction. Initial data cleaning can greatly improve the downstream ML performance and is the utmost important step prior to the commence of any ML tasks [38]. For example, some enzymes undergo various post-translational modifications, such as signal peptide cleavage, before becoming mature or functional, thus the modification process should be taken in consideration in data processing to potentially increase the prediction accuracy. Automatic data cleaning may miss some features regarding this aspect, due to the massive nature of the dataset. Even in AlphaFold Protein Structure Database (https://alphafold.ebi.ac.uk/), the predicted structures of annotated fungal laccases, such as *Or*Lac1 (UniProt: I1W1V7), still contain extended N-terminal signal peptides with low model confidence score. Manual systematic literature review still plays a critical role in data preparation, yet it is very labor-intensive. Automation or semi-automation of literature searching by involving computational methods [39] may speed up the process. Alternative promising solution to mitigate the limits of using only small dataset may involve a meta-learning strategy [3], with a nested optimization including training on a large set of noisy examples in the inner loop and a small set of trusted examples in the outer loop, thus suppressing the impact of the noise in the larger training dataset.

In computational biology, each ML algorithm has its pros and cons on a specific training dataset. For example, random forest is a popular method due to several advantages over other algorithms, such as dealing with high-dimensional feature space, a small number of samples, and complex data structures [40]. Random forest learns how important each feature is to the prediction. Individual decision trees are human readable, allowing interpretation of how a decision is made. Moreover, it is less sensitive to feature scaling and normalization making it easier to train and tune, as seen in our study here. Nevertheless, the model is less appropriate for regression and many decision trees are hard to interpret [41]. Gradient boosting has the same advantages, but it can struggle to learn underlying signal if noise is present, and it also is less appropriate for regression. Therefore, by combining the prediction results from various models can lower the weight of each model bias and give more accurate predictions (Fig. 4). A more systematic balancing system taking the advantage of individual models may be developed in the future to get a better overall score for the prediction responses. It is noteworthy that ML models are trained on existing datasets, hence may struggle to discover completely novel enzymes or new enzymatic properties [3, 42]. For predictions on divergent or unprecedented enzyme families, experimental data collection may still be inevitable. With the recent breakthrough of large language models, they may offer promising approaches to enhance to decode enzyme catalysis and uncover novel enzymes [3, 43].

In recent years, advances in deep learning have led to remarkable improvement in predicting protein properties from amino acid sequences [44]. A recent unrefereed preprint also proposed a language model-based semi-supervised algorithm EpHod in predicting enzyme optimal pH from a broader range of protein sequences that were numerically represented using various methods [45]. In our study, the numerical representation of protein sequences using canonical amino acid composition only generated a 20-dimensional feature vector. Higher dimensional numerical representations of the protein sequences, such as per-residue embeddings from ESM-1v [46], may also be considered to distinguish proteins with different primary structures but identical amino acid composition. Furthermore, as shown in Fig. 2 and Fig. S2, it is important to incorporate any relevant features in addition to sequence-based ones in building a high-quality training dataset from only limited numbers of instances. For example, a more precise classification could be considered as a feature when predicting pH optimum from a wide range of enzymes beyond the AA1_1 class.

For enzymes, such as laccases, pH optimum is critical, as the environmental pH affects drastically their catalytic efficiency and stability, and hence determines the applicability of the enzymes in different industrial processes [47]. As the protein surface is the interface through which a protein senses the external environment, the composition of charged, polar, and hydrophobic amino acid residues is crucial for the stability and activity of the protein [48]. Although the explicit mechanism by which surface amino acids contribute to the enzyme’s adaptation to acidic or alkaline environments remains ambiguous, surface charge is often believed to play a role. Previous protein engineering studies indeed lowered the pH optimum by increasing negative surface charge of the enzyme [49, 50], while studies reducing negative charges of surface residues clearly shifted the pH optimum to more alkaline values [51, 52]. In our study, it was observed that alkaline laccases from basidiomycete fungi possess significantly more non-polar aromatic residues on their surface than acidic laccases (Fig. 6C). Aromatic amino acids contribute to the hydrophobic interactions that stabilize the enzyme’s tertiary structure and are important for protein folding, protein–protein interaction, and ligand binding [53]. Thus, although the absolute numbers of *Ln*Lccs surface aromatic residues are low compared to other residues, this subtle difference may be sufficient to contribute to their alkaline pH optimum, which could be recognized by the ML models. Additionally, altering pK_a_ values of residues in the substrate binding site often showed significant impact on the enzyme’s pH optimum [54, 55]. However, the pK_a_ can be influenced by many complex and interrelated mechanisms, such as changes in the electrostatic environment [56], protein conformation [57] and solvent accessibility [58], which can be influenced to some elusive extent by the surface amino acid composition. Therefore, future ML models for the prediction of pH optimum focusing on the use of surface amino acid and individual pK_a_ values and spatial locations of the residues in the binding cavity as input data might generate more accurate prediction results.

It is worth mentioning that both *L. nuda* laccases have distinguished pH preferences for the oxidization of 2,6-DMP and ABTS (Fig. 7A, S4). This observation aligns with many studies on the discovery of alkaline laccases [52, 59–62], which showed substrate-specific pH optimum. The ABTS assay relies on the stability of ABTS radicals, which rapidly degrade as pH increases [63]. In contrast, the oxidization of 2,6-DMP by laccases can be measured over a wider pH range [12, 59]. Therefore, for predicting alkaline laccases, the docking possibilities and pH optimum of the training dataset were only measured for 2,6-DMP, which were then picked up by the ML models to generate predictions of the pH optimum. As shown in Fig. 3, the affinity of 2,6-DMP to the enzymes indeed exhibited certain attention weights from models like RFSRC and LGBM. Yet the overall weights of the biochemical features were still low, compared to the features representing amino acid composition. For future ML models, incorporating binding information on a broader substrate range during feature extraction may improve the model performance.

Additionally, both *Ln*Lccs have two distinct pH optima for 2,6-DMP oxidization with 1 and 1.5 pH units apart (Fig. 7A, B). Having double pH optima is not unusual property of enzymes [64]. The theory behind the mechanism of enzymes’ double pH optima may be rooted from the two distinct conformations of the enzyme, which are reversibly convertible at different pH values [65]. Therefore, the subsequent formation of enzyme–substrate complexes by the two ionic species of the enzyme are uniquely different. It is also possible that an endogenous ampholyte inhibitor, such as metal ions in the buffer, inhibits the enzyme–substrate binding, causing a dip at the enzyme’s theoretical pH optimum and splitting the optimal activity peak into two [64]. Nevertheless, possessing double pH optima cannot deny the fact that both *Ln*LccA and *Ln*LccB oxidize the phenolic substrate 2,6-DMP the best at alkaline conditions.

While small training data are often the bottleneck in biological studies, our study as a proof of concept shows that small but high-quality training dataset of only 55 instances with careful selection of appropriate models is sufficient to accomplish the massive prediction of enzyme pH optimum on a test dataset of 1964 instances. The correct prediction of alkaline laccases from basidiomycete fungal genomes in this work displays the potential of ML in identifying enzymes candidates with desirable properties from vast genomic datasets. The selection of *Ln*LccA and *Ln*LccB for biochemical characterization, based on their moderate cophenetic distances to known alkaline laccases and distinct prediction responses, demonstrates the application of computational predictions to streamline experimental validation efforts.

Computer and ML-aided approaches have been used in enzyme rational design [66], targeted identification from metagenome [67, 68]. Yet none of those earlier methods have incorporated extensive downstream analyses to validate the ML-predictions. Only until very recently, ML prediction was applied to predict enzyme kinetic parameters, accompanied with intensive downstream applications and wet-lab experimental validations [14]. Researchers are envisioning ML as a powerful tool to complement the traditional functional design of enzymes through directed evolution [69]. On the cusp of the artificial intelligence revolution, our extended approach, based on but not limited to traditional ML models, indicates the importance of considering the prospective application in which the trained models will be used in practice to ensure optimal performance. This is shown by computational characterization, including solubility profiles and structural predictions, which provide a molecular basis for the observed pH optima and offer insights into the structural determinants of enzyme activity. A future model, integrating possible computational characterization of selected candidates would further streamline the ML models by supporting their prediction results automatically.

Our findings contribute to the understanding of enzyme adaptation to environmental pH conditions, offering insights into the design of enzymes with tailored pH optima for industrial applications. Future research should focus on expanding the dataset, refining model predictions, and exploring the structural basis of enzyme function to further unlock the potential of laccases in various biotechnological applications with large impact for future lignocellulosic biorefineries, biocatalysis and environmental remediation, among others. Explicit analysis of protein three-dimensional structures rather than primary protein sequence in ML or deep learning may explore the subtle ties between enzyme structure and function for a precision discovery of novel enzymes and contribute to better understanding of enzymatic mechanism.

## Conclusion

As a proof of concept, our study not only demonstrates the efficacy of an ML-integrated approach in precise prediction of enzyme pH optimum, but also highlights the tremendous potential of computational tools in the discovery and characterization of potential biocatalysts for industrial applications. This approach integrates computational predictions as the core, supplemented with comprehensive in silico characterization and robust experimental validation. By curating data from other enzyme classes, this strategy can be a useful tool for metagenomic mining of any enzymes, thus ultimately facilitating the discovery and targeted design of enzymes with enhanced catalytic properties to advance biotechnology applications.

## Supplementary Information

Supplementary Material 1.

Supplementary Material 2.

## Data Availability

All data generated or analyzed during this study are included in this published article and its Supplementary files. Data that are indicated as “Data not shown” in the article will be made available on request.
