# Supplementary material for: Discovery of alkaline laccases from basidiomycete fungi through machine learning-based approach
Source: Biotechnol Biofuels Bioprod. 2024 Sep 11;17:120. doi: 10.1186/s13068-024-02566-6 (PMC11391777; doi:10.1186/s13068-024-02566-6)
Supplement: Supplementary file 2 — Supplementary Material 2. [file 13068_2024_2566_MOESM2_ESM.docx]

**Supplementary figures**

**Discovery of alkaline laccases from basidiomycete fungi through machine learning based approach**

Xing Wan^1, *^, Sazzad Shahrear^1^, Shea Wen Chew^1^, Francisco Vilaplana^2^, Miia R. Mäkelä^1, 3, *^

^1^ Department of Microbiology, Faculty of Agriculture and Forestry, University of Helsinki, Biocenter 1, Viikinkaari 9, 00790 Helsinki, Finland

^2^ Division of Glycoscience, Department of Chemistry, School of Engineering Science in Chemistry, Biotechnology and Health, KTH Royal Institute of Technology, AlbaNova University Center, Roslagstullbacken 21, 11421 Stockholm, Sweden

^3^ Department of Bioproducts and Biosystems, Aalto University, Kemistintie 1, 02150, Espoo, Finland

^*^ Corresponding authors.

*E-mail addresses*: xing.wan@helsinki.fi (XW), miia.makela@aalto.fi (MRM)

**Summary**

This file includes four figures.

**Fig. S1.** Putative substrate binding cavity of *Panus rudis* (JGI Protein ID: 1594824) laccase mentioned in Materials and Methods, section of Feature extraction and selection.

**Fig. S2.** Permutation feature importance is used in Results, section of Model interpretation, as the alternative method to SHAP.

**Fig. S3.** Heatmap is used in Results, section of Prediction of laccases with an alkaline pH optimum to narrow down the potential alkaline laccases for further analyses.

**Fig. S4.** Effect of pH on the activity on ABTS of the recombinant *Lepista nuda* laccases, mentioned in Results, section of Biochemical characterization.


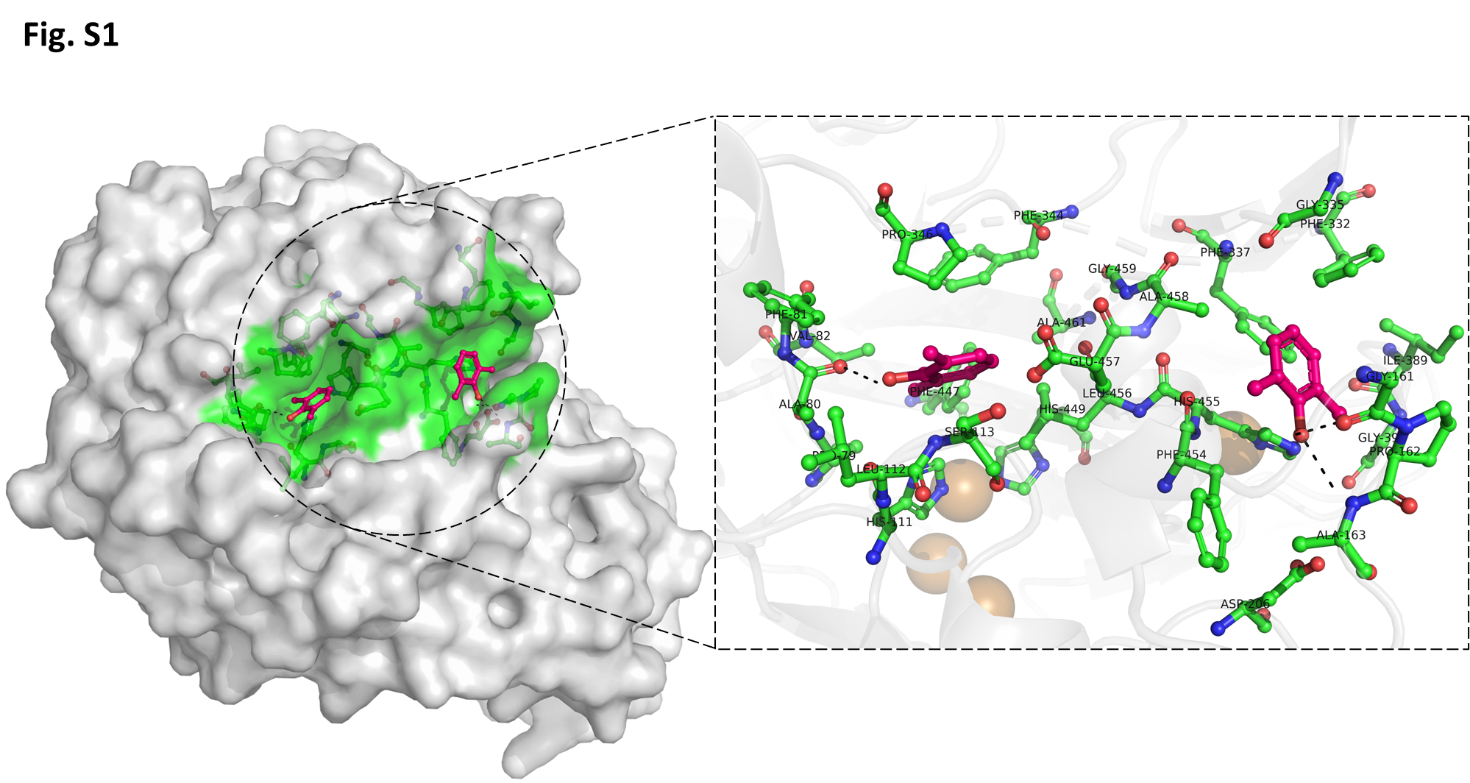


**Fig. S1.** Putative substrate binding cavity of *Panus rudis* (JGI Protein ID: 1594824) laccase. Reference laccase 2HRG from *Trametes trogii* was superimposed with *P. rudis* laccase (JGI Protein ID: 1594824, grey surface and cartoon) to locate the copper centers (brown spheres). Substrate docking was performed using SwissDock with default settings, and only docking positions close to copper centers were analyzed further. Visualization was carried out in PyMol. Model substrate 2,6-DMP is shown in magenta sticks; left, binding position with the least energy while forming hydrogen bonds; right, binding position with the second least energy while forming hydrogen bonds. Residues within 5 Å from the substrate molecules are shown in green sticks and labeled with the amino acid 3-letter abbreviation and location. Black dashed lines indicate the hydrogen bonds between the substrate molecules and their binding residues.


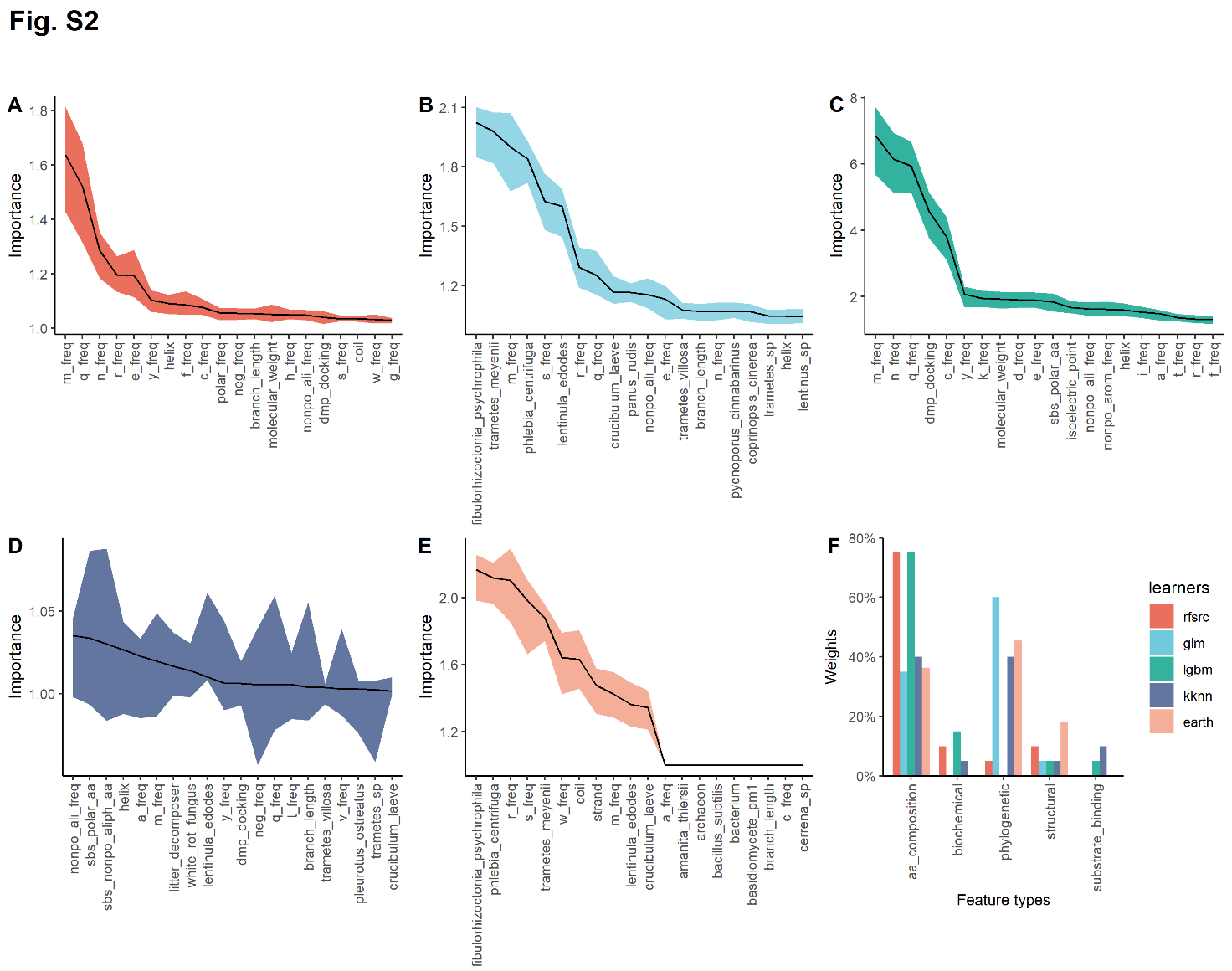


**Fig. S2.** Permutation feature importance for the four tuned models (A) RFSRC, (B) GLM, (C) LGBM, (D) KKNN, and one native model (E) EARTH, and the occurrences of each feature types on the prediction decisions (F).


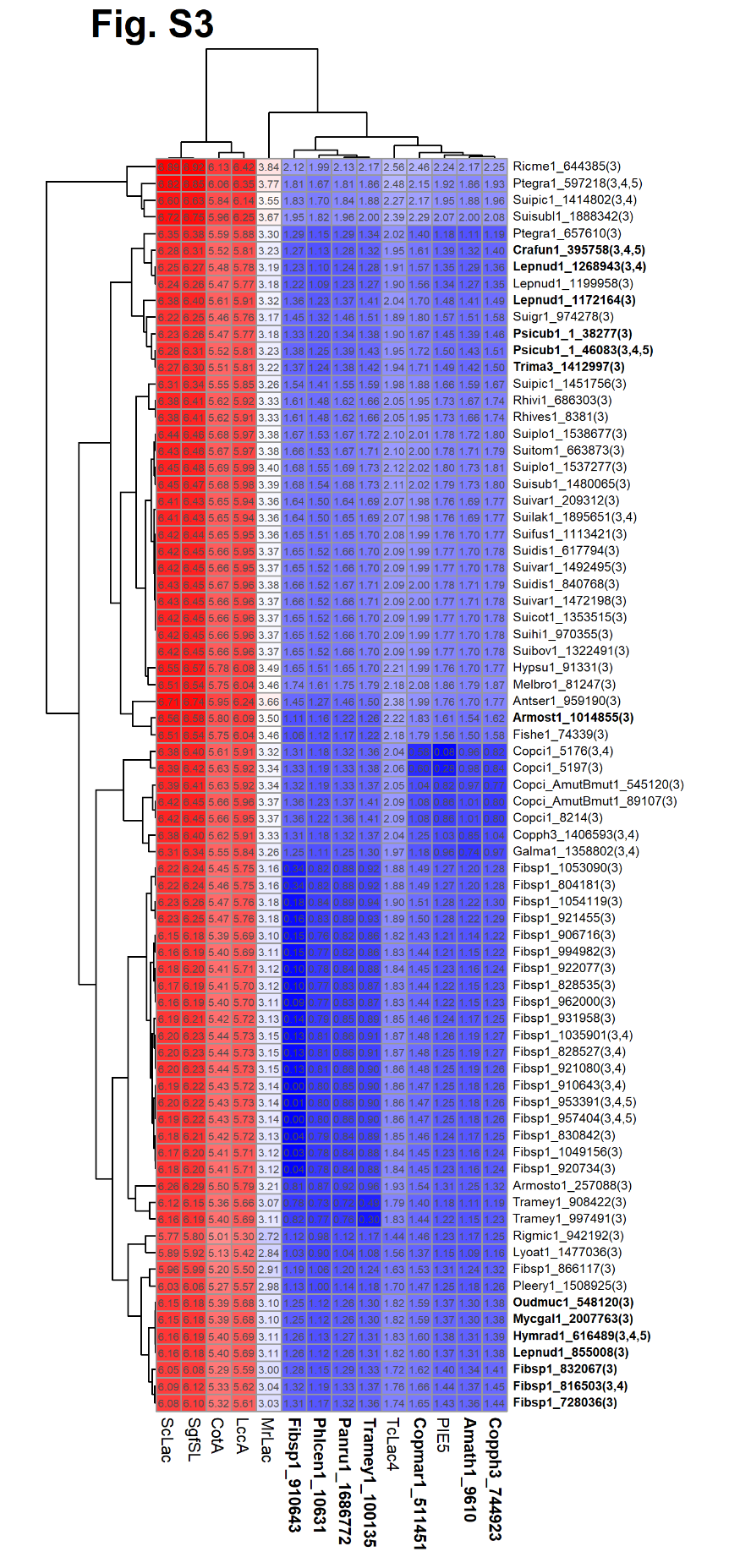


**Fig. S3.** Heatmap showing the cophenetic distances between the reference laccases (at bottom) and the 75 candidate laccases (on the right) which predicted pH optimum is over pH 7.0. The number of models giving consensus prediction responses are shown in brackets. The smaller subset of 14 candidates with moderate cophenetic distances to known extreme alkaline laccases but longer distances to known neutral laccases are shown in bold. The reference neutral and alkaline basidiomycete laccases are also shown in bold. Laccases information can be found in Table S1.


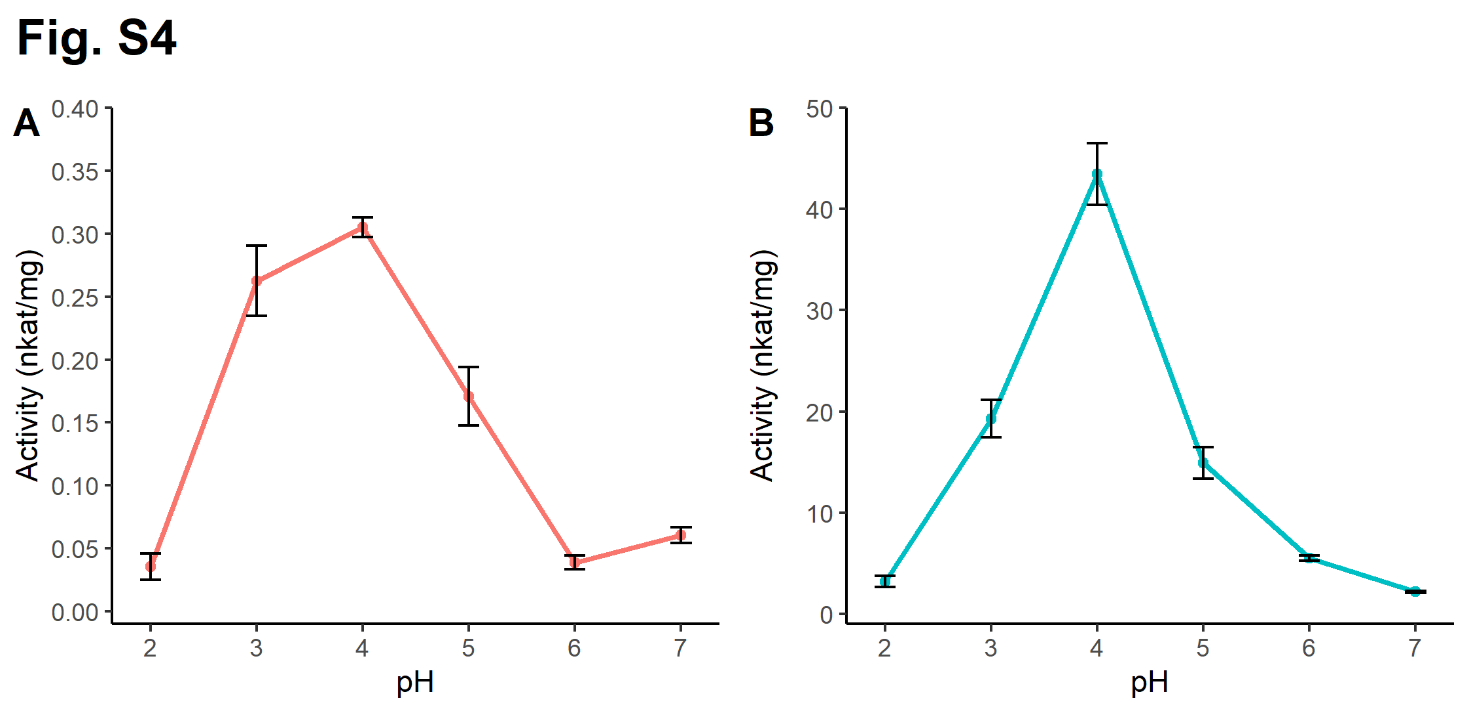


**Fig. S4** Effect of pH on the activity of recombinant *Lepista nuda* laccases towards ABTS. (A) *Ln*LccA and (B) *Ln*LccB. Note that the specific activities of *Ln*LccA and *Ln*LccB are presented in different scales. The error bars represent standard deviation from the triplicate measurements.
